# Stress perfusion CMR in hypertrophic cardiomyopathy: comparison with late gadolinium enhancement

**DOI:** 10.1186/1532-429X-16-S1-P324

**Published:** 2014-01-16

**Authors:** Adriana Villa, Nuno Bettencourt, Niloufar Zarinabad, Rocio Hinojar Baydes, Eike Nagel, Amedeo Chiribiri

**Affiliations:** 1 Cardiovascular Imaging, King's College London, London, UK; 2 Cardiology, Centro Hospitalar de Gaia/Espinho, Porto, Portugal

## Background

Hypertrophic cardiomyopathy (HCM) has a wide clinical spectrum. An important subgroup of patients are at risk of major complications and sudden cardiac death. However risk stratification in these patients is still a challenge. The degree of hypertrophy and myocardial fibrosis are important predictors of prognosis. There are several papers showing the possibility of areas of abnormal left ventricular (LV) perfusion in patients with HCM. Some studies focused on stress-induced perfusion defects (Petersen SE, et al. Circulation 2007;115:2418-2425), demonstrating the presence of epi-endocardial gradients of perfusion in the LV due to microvascular dysfunction. Some other studies (Matsunaka T MRSM 2003;2:61-9 - Melacini P, Int J Cardiol 2008;128:364-73) have focused the attention on LGE-related rest perfusion abnormalities. These are due to areas of microvascular disruption and do not contribute necessarily towards the LV ischemic burden. Mixed defects are also possible. The aim of the study was to analyse in retrospective a group of patients with HCM that underwent stress perfusion and LGE-CMR and to describe the prevalence of perfusion defects of each type.

## Methods

This study included 33 consecutive patients with HCM. The images were analysed by consensus of 2 expert CMR readers. The results were reported on segmental basis, indicating the presence or absence of perfusion abnormalities on stress perfusion scans, the presence of a perfusion gradient, the presence of areas of scar related perfusion abnormalities or the presence of mixed perfusion defects.

## Results

Hypertrophy, LGE and perfusion defects involved more frequently the inter-ventricular septum. No patients in this group had severe LV hypertrophy (max LV thickness > 30 mm). LGE had a prevalence of 76%, while perfusion abnormalities with a gradient pattern were seen in 87% of patients in at least 1 LV segment. Perfusion abnormalities were present with a pure gradient pattern in 55% of the LV segments. LGE-correlated abnormalities were seen alone in 15% of the segments. Mixed perfusion abnormalities were seen in 30% of the segments.

## Conclusions

Our results demonstrate that CMR is a useful tool to identify perfusion abnormalities in patients with HCM. Stress-induced perfusion defects are frequently present in patients with HCM also in the absence of severe LV hypertrophy. The intrinsic high spatial resolution allowed by perfusion and LGE-CMR enables the differential diagnosis of perfusion abnormalities indicative of microvascular dysfunction, contributing to the LV ischemic burden, and those related to the presence of scar. Combined perfusion and LGE-CMR has the potential to increase the accuracy of quantification of the true LV ischemic burden in patients with HCM.

## Funding

The Centre of Excellence in Medical Engineering is funded by the Wellcome Trust and EPSRC under grant number WT 088641/Z/09/Z. The authors acknowledge financial support from the Department of Health via the National Institute for Health Research (NIHR) comprehensive Biomedical Research Centre award to Guy's and St Thomas' NHS Foundation Trust in partnership with King's College London.

